# Gastric Cancer: Innovations in Screening, Diagnosis and Treatment

**DOI:** 10.3390/jpm13010127

**Published:** 2023-01-08

**Authors:** Li Liu, Wenhong Deng

**Affiliations:** Department of Gastrointestinal Surgery, Renmin Hospital of Wuhan University, Wuhan 430060, China

Gastric cancer (GC) is an aggressive and heterogeneous malignancy that is one of the leading cancers in the world and an important global health problem due to its overall high prevalence and high mortality rate. Less than a century ago, gastric cancer was the most common cancer in the United States and worldwide. Although the incidence of stomach cancer has declined worldwide over the past century, it still remains a major global killer [1].

Early screening for gastric cancer can be used in large populations (mass screening) or in high-risk individuals (opportunistic screening). Although the value of screening for gastric cancer in large populations remains controversial, it has been offered in some countries with high GC prevalence, such as Japan, Venezuela, and Chile [2]. In contrast, in countries with a low incidence of GC, such as the United States, this strategy is expensive and unjustified. In low-risk areas, only high-risk populations with certain diseases may benefit from GC screening, including older adults with chronic gastric atrophy or pernicious anemia and patients with gastric polyps, partial gastrectomy, familial adenomatous polyposis, and hereditary non-polyp colon cancer [3]. Several serum markers have been proposed to detect patients at risk for GC. As atrophic gastritis develops, serum pepsinogen I (PGI) concentrations decrease, while serum pepsinogen II (PGII) levels remain relatively constant. Therefore, the serum PGI/II ratio can be used as a marker for future gastric cancer development. Several studies have shown that the PGI/II ratio can be used as a continuous marker: the lower the PGI/II ratio, the higher the risk of GC. Combining H. pylori serology and serum pepsinogen concentrations may help to better predict the development of GC. It has been suggested that the combination of a low PGI (or PGI/II ratio) with a negative H. pylori antibody indicates the highest risk [4], as it may indicate very severe atrophy leading to a decline in H. pylori populations in the stomach. Chronic inflammation and atrophic gastritis caused by H. pylori infection may reduce gastric hunger production, so low serum gastric hunger may indicate a higher risk of gastric cancer. Gastrin 17 or anti-gastric lining cell antibody (APCA) may also be used as serum markers for gastric cancer [5]. In addition, examination of the gastric mucosa using barium swallow fluoroscopy or endoscopy is a common screening tool, and upper gastrointestinal endoscopy is the gold standard for the diagnosis of gastric cancer. This technique has a high detection rate and is widely used for GC screening in high-risk areas, such as Japan [6].

The basic diagnostic tools for gastric cancer include endoscopy and imaging, which are used for the qualitative localization and staging of gastric cancer. Others include physical examination, laboratory tests, biopsy of metastases, and diagnostic laparoscopic exploration and evaluation of abdominal lavage fluid. CT of the thorax, abdomen, and pelvis is the basic tool for pre-treatment staging, while MRI, laparoscopic exploration, and PET are used as alternative tools for CT suspicion of liver metastases, peritoneal metastases, and systemic metastases, respectively. The imaging report provides a description of the signs involved in cTNM staging and gives a staging opinion. Histopathological diagnosis by endoscopic biopsy is the basis for the confirmation of the diagnosis and treatment of gastric cancer. It provides the pTNM staging of gastric cancer, which provides the necessary histopathological basis for clarifying the histological type of gastric cancer, comprehensively assessing the progression of gastric cancer, judging the prognosis of patients, and formulating targeted, individualized treatment plans [7]. Currently, molecular staging of gastric cancer based on the HER2 expression status of tumor tissues is the basis for selecting anti-HER2-targeted drug therapy, and HER2 testing is necessary for all cases with pathologically confirmed adenocarcinomas of the stomach or esophagogastric junction. MSI/dMMR status is recommended for evaluation in gastric cancer tissues. The application of next-generation sequencing (NGS) and liquid biopsy in gastric cancer is currently in the exploratory and data accumulation stage [8].

Treatment selection for gastric cancer should be based on the clinical stage. For early stage gastric cancer, endoscopic treatment, namely endoscopic mucosal resection (EMR) and endoscopic submucosal dissection (ESD), is preferred. For patients who are not suitable for endoscopic treatment, open surgery or laparoscopic surgery can be performed. The treatment of patients with advanced gastric cancer without surgical opportunities is a real challenge, and it is now recognized that a combination of systemic drug therapy and local therapies, such as palliative surgery, radiotherapy, radiofrequency ablation, peritoneal perfusion, and arterial interventional embolization and perfusion, should be adopted. Unfortunately, the complete cure rate for GC has not progressed enough in the last 20 years, and we are still unable to save the lives of >70% of patients. This highlights the need to enhance all means of diagnosing GC at an early and curable stage, on the one hand, and to work harder to develop new and more effective drugs for advanced GC, on the other. The cornerstone treatment for metastatic GC remains cytotoxic chemotherapy, as most targeted therapies are effective in other GI cancers, such as colorectal cancer (bevacizumab, cetuximab), but seem to be ineffective in GC. Positive results were obtained only with the anti-HER2 drug trastuzumab (available as first-line therapy) and the anti-angiogenic drug ramucirumab (available as second-line therapy) [9]. Recent interest in the potential efficacy of immune checkpoint inhibitors has brought some hope that monotherapy with the immune checkpoint inhibitor nivolumab (PD-1 monoclonal antibody) can be used in the treatment of advanced gastric cancer. The future challenge will be using all available histological and molecular markers to identify patients who are likely to benefit from specific treatments [10].

In conclusion, in the field of GC, we should move toward better detection of early cancers, more appropriate monitoring of gastric precancerous lesions, more precise identification of patients likely to benefit from specific treatments, and a better understanding of gastric carcinogenesis to identify new therapeutic targets for advanced GC.
